# Development of a Remote Displacement Measuring Laser System for Bridge Inspection

**DOI:** 10.3390/s22051963

**Published:** 2022-03-02

**Authors:** Kyung-Hwa Kim, Hwee-Kwon Jung

**Affiliations:** Intelligent Photonic IoT Research Center, Korea Photonics Technology Institute, 9, Cheomdan Venture-ro 108beon-gil, Buk-gu, Gwangju 61007, Korea; jhk0946@kopti.re.kr

**Keywords:** laser system, laser sensor, displacement measurement, safety inspection, bridges

## Abstract

Measuring displacement is essential for assessing the safety of bridges. Non-contact sensors such as vision sensors can precisely measure displacement but may be expensive or incapable of micro-scale measurement at a low cost, unlike contact displacement sensors, which are economical but challenging to install. This study proposes an economical, remote non-contact sensor system. The system comprises a laser beam transmitter and a light receiver, deriving the displacement based on the position where the laser beam is irradiated to the light-receiving surface. To measure this, the light receiver was installed at the measurement point and included a wireless communicator to transmit the displacement data. A displacement experiment was conducted to evaluate the performance. The results confirmed that precise displacement measurements were possible at a resolution of 100 µm. For bridge load tests, a light receiver under a bridge was installed, laser beams irradiated to the light-receiving surface from a distance, and the displacement was measured for each test and compared with the values measured by a conventional contact sensor. The results were highly consistent with those of the existing sensor, indicating that the proposed sensor system applies to bridge loading tests and the safety diagnosis for various structures.

## 1. Introduction

Among the measurements used to diagnose the safety of bridges, displacement is the most reliable. This is because it is related to the overall stiffness, which is closely related to the resistance under various scenarios (environmental factors such as earthquakes and typhoons, vehicle traffic, etc.).

Contact sensors such as linear variable differential transformers (LVDTs), which are economical and enable precise measurement, are mainly used to measure displacement. However, as they determine the position change of the measurement point regarding a fixed point, they cannot be used if there is a river, sea, road, or railroad under the bridge or if the bridge is significantly high. Moreover, their installation requires extensive labor and long periods of traffic control, and because large structures such as bridges necessitate multiple sensors, wiring management issues also arise.

Researchers have developed various non-contact measurement techniques to address the limitations of existing contact sensors, such as GPS [1,2,3,4], laser Doppler velocimeters (LDVs) [5,6,7], radar interferometers [8,9,10,11], and robots or unmanned aerial vehicles (UAVs) [12,13,14]. Among these, vision sensor-based methods have received attention from many researchers because they are economical, enable remote simultaneous multi-point measurement, and are easy to install [15,16,17,18,19,20,21]. Pan et al. used these and the digital image correlation method to measure bridge displacement [19]. Their technique did not require the installation of additional markers on the bridge and could measure sub-pixel motion, thus enabling high-precision measurement compared to existing vision sensor-based methods. However, these methods are difficult to use in environments with insufficient light (e.g., at night). Given that bridge safety is typically inspected in the evening when it is easy to control traffic, a method that can be used even in low-light settings is necessary. Hence, researchers applied the vision sensor concept using LEDs as the active markers. Using this method, Tian and Pan developed a vision sensor-based bridge deflection measurement system and monitored a bridge to verify the system [20,21]. Although vision sensors have numerous advantages, it is difficult to measure as precisely as contact or other non-contact sensors. The development of diverse image processing techniques has enabled the measurement of sub-pixel motion; however, sufficient light is necessary for precision. Moreover, although large bridges can be measured with images because their deflection is mostly over 10 mm, generally, small and medium bridges exhibit displacement within 3 mm in areas showing the most considerable deflection, making it challenging to apply image-based techniques to general bridges.

GPS-based methods are easy to use and can measure displacement in all directions but have low precision, while radar interferometers can perform remote precision measurements but are unstable. This is because the measured value changes sensitively according to the wavelength. Meanwhile, robots and UAVs measure displacement using a variety of sensors, such as GPS, radar, and vision sensors.

Precise optical sensors mainly comprise laser-based sensors. LDVs can be used during the day and at night and are capable of remote precision measurement, but they are expensive and can measure only a single point at a time. Although researchers have developed an LDV capable of multi-point measurement using multi-modulated laser light, this technique becomes more expensive due to the many optical modulation parts involved [22]. Hence, precise and economical laser-based sensors have been developed and applied to bridges.

In a laser-based sensor, a position-sensitive detector (PSD), charged couple device (CCD), and complementary metal oxide semiconductor (CMOS) are used as the light-receiving elements. These sensors use a triangulation measurement system. The laser beam is condensed by the lens and directed onto the object. The laser beam reflected from the object is condensed onto the light-receiving element by the receiving lens. If the object’s position (the distance to the measuring device) changes, the image-formation positions on the PSD differ, changing its outputs. Regarding CCD and CMOS, the amount of light on individual pixels changes. The displacement is converted into a distance when a spot beam that reflects off the object’s surface is projected onto the light-receiving element.

Zhao et al. [23] developed a laser-based sensor using a laser and camera to measure the displacement of a bridge. The laser was installed so that it irradiated to a plate installed on the measurement point of the target structure, while the camera estimated the laser beam’s position on the plate to derive the displacement. However, the accuracy varies with the image resolution, camera angle of view, measurement distance, camera frame per second (FPS), and environmental factors; therefore, additional research is needed to apply this technique to bridges.

For bridge inspection and monitoring, several long-distance sensors including the radar interferometer, LDV, camera-based sensor, and laser-based sensor are used; their characteristics are presented below.

**Radar interferometer**: Precise measurement is possible, but when the wavelength of the light source is changed due to the external environment, the accuracy decreases. Furthermore, an error may occur in the displacement measurement process through the phase difference between the reference light and the reflected light.

**LDV**: Precision measurement is possible, but it is expensive. Therefore, it is difficult to apply to bridge monitoring and the measurement is limited to a short range.

**Camera-based sensor**: Simultaneous multi-point measurement is possible, but it cannot be applied in dark environments, and precise measurement is difficult.

**Laser-based sensor**: Precise measurement is possible and economical, but precision decreases as distance increases because the triangular method is used. Moreover, the installation space and place are limited for measuring the vertical movement of the bridge because a reflector is used.

Therefore, it is necessary to develop a sensor suitable for measuring the displacement of a bridge while overcoming the disadvantages of the above-mentioned sensors. Since the bridge covered in this study was not a long-span bridge, it had minute displacement and only one representative point needed to be measured; the sensor was developed by selecting a laser-based sensor type.

Compared to the laser-based sensor techniques, the technique developed in this study does not use any reflector. In the technique, the light receiver is directly attached to the structure without a reflector. Regardless of the distance, the same measuring precision can be secured if the laser beam is sufficiently focused on the light-receiving surface and the vertical movement of a bridge can be measured both under the bridge and on the side.

This study introduces a sensor system capable of remote precision measurement applicable to general but not long-span bridges. In detail, this paper first explains the sensing system, and then describes the performance evaluation conducted through a lab-scale experiment. Finally, the results of applying the system to a bridge and the system verification are explained.

## 2. Laser and PSD-Based Remote Displacement Measuring System

**Figure 1:**
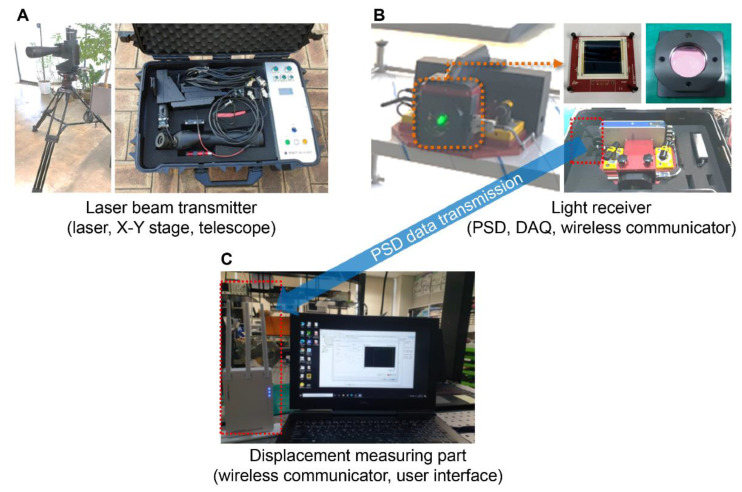
System configuration of the remote displacement sensor. (**A**) Laser beam transmitter including laser, X-Y stage and telescope, (**B**) Light receiver including PSD, DAQ and wireless communicator, (**C**) Displacement measuring part including wireless communicator and user interface).

The remote displacement measurement system developed in this study comprises a laser beam transmitter, light receiver, wireless data transmitter, and displacement measuring part, as shown in Figure 1.

**Figure 2:**
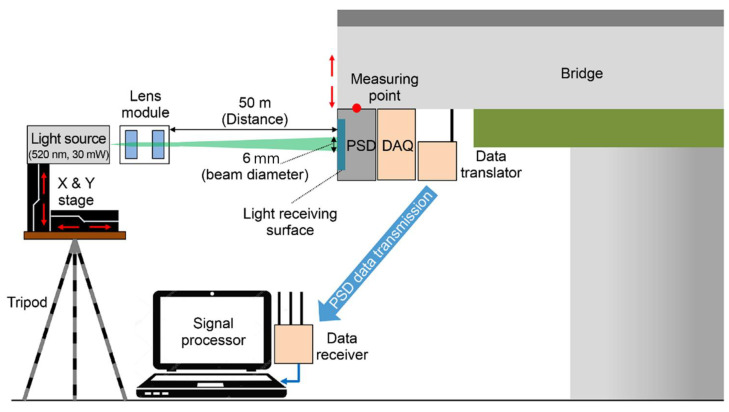
Concept of the sensor application to a bridge.

The laser beam transmitter comprises a tripod, laser module, automatic zero-point adjustment device to begin measurement from the center of the light-receiving surface, and a short telescope to confirm the remote laser beam. For the laser light source, a 520 mm continuous wave laser with visibility and output of 30 mW was selected so that the system could efficiently receive light even from a long distance. To enhance precision, the optical system was designed with a beam size of 6 mm at 50 m and 10 mm or less at 100 m. Figure 2 shows the concept of the sensor applied to a bridge.

The light receiver was developed to achieve a displacement detection distance of ±20 mm at 0.1 mm resolution. A two-dimensional PSD was used for the light-receiving element (SiTek, 2L45_SU24). Moreover, considering the usage environment of bridges, a band-pass filter was inserted to filter out ambient light other than the laser light source. In a PSD, which is an optical sensor used to detect position, when light is incident on the light-receiving area, photoelectrons are generated, and output is in a photoelectric current form. The displacement can be calculated according to the load of the bridge by applying the principle that the current value varies with the incident position. As the system uses a two-dimensional PSD, it can measure vertical and horizontal displacement. We designed and fabricated a circuit for position signal detection and used the NI9233 device to collect the displacement data.

During displacement measurement, the PSD derives the position on the light-receiving surface where the maximum signal intensity is measured. Although the diameter of the laser beam was 6 mm on the light-receiving surface during long-distance measurement, the shape of the signal recognized by the light-receiving surface is convex as with a 2D Hanning window, and only the position of the maximum value point in the signal. Therefore, the resolution of 0.1 mm could be conserved.

A Wi-Fi module (NI cDAQ™-9191, Ethernet, and 802.11) was used to transmit the data measured by the light receiver wirelessly. A PC receives the data through a wireless router, and calculates and displays the displacement.

This system can precisely measure displacement even from a long distance, has a simple configuration, and is highly robust, making it suitable for use in diverse environments. Furthermore, because it is installed under the bridge, it can be used during the day and at night.

## 3. Accuracy Validation of the Remote Displacement Measuring System

We performed a lab-scale experiment to verify the displacement measurement accuracy of the developed system.

**Figure 3:**
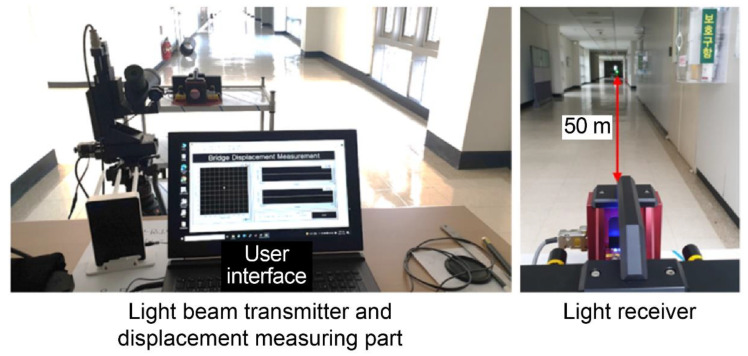
Lab-scale experiment setup for accuracy validation.

**Figure 4:**
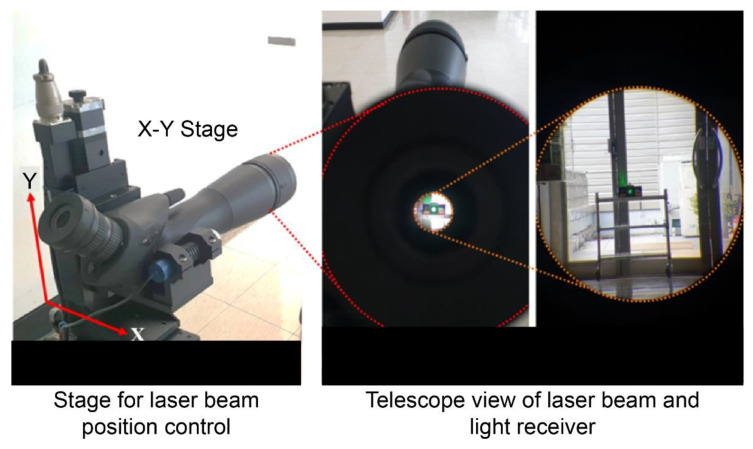
Motorized Stage along the *X*- and *Y*-axes and telescope of light source part.

As shown in Figure 3, we first ensured a 50 m distance between the light receiver and laser beam transmitter, and then adjusted the latter to the center of the light-receiving surface. Subsequently, as shown in Figure 4, it was moved in each direction by controlling the *X*- and *Y*-axes directions, and the displacement was measured according to the control. The displacement was continuously measured at a sampling rate of 30 Hz. An average filter was also applied to suppress noise from ambient light. Since the signal measured in PSD had a large signal variation due to noise, a signal processing technique called average filtering was applied to stabilize the signal. In this technique, the average value of the previously measured signal is derived to represent the current value. At the time of the laboratory-scale test, the average value was derived after measuring the previous 10 signals and indicated as the current value.

For simulating displacements in *X*- and *Y*-axes directions, the Motorized Stage, which is a mechanical device for generating linear movement, was used. As the Motorized Stage was controlled at a low speed, similar to a quasi-static state in the experiment, the control values and stage position values matched. The Motorized Stage was configured to wait at the initial position for 3.6 s, move to the set position within 2 s, wait again for 1 s, and then return to the original position.

**Figure 5:**
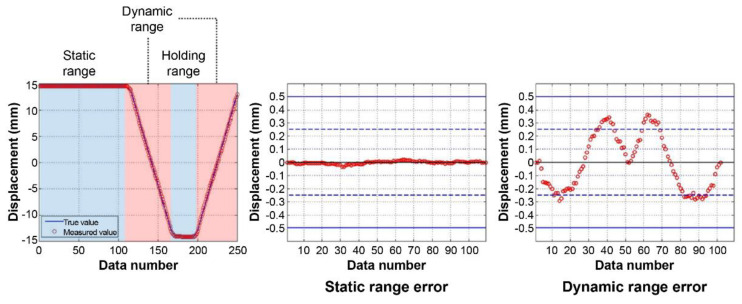
*X*-axis movement signal comparison (stage control vs. measured displacement) and errors of statics and dynamics ranges.

**Figure 6:**
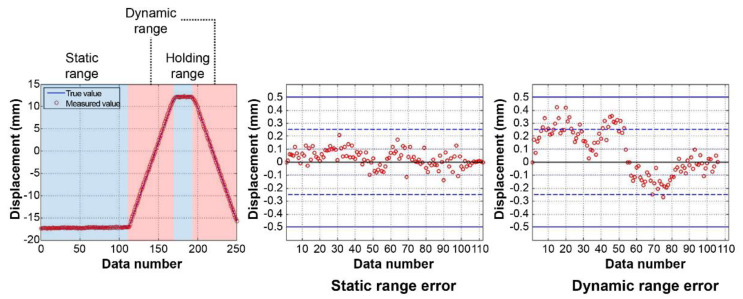
*Y*-axis movement signal comparison (stage control vs. measured displacement) and errors of statics and dynamics ranges.

To verify the displacement measurement accuracy, the Motorized Stage control and measured displacement values were compared. Figure 5 shows the comparison results when the Motorized Stage was moving along the *X*-axis, and Figure 6 depicts when it was moving along the *Y*-axis.

To analyze the error according to state, we divided the data into (i) a static range where the stage statically waited, and (ii) a dynamic range where its movement was controlled. Regarding the *X*-axis control, the error was significantly small when statically waiting. The mean and standard deviation of the errors 0.01 mm and 0.01 mm, respectively, and the maximum error was 0.03 mm. Conversely, an error occurred while moving the light source because the lower frame and tripod moved from the recoil when the Motorized Stage was controlled. As shown in the error values, the signal values are created in the same direction as the Motorized Stage’s movement, forming a sine wave shape. Hence, the mean and standard deviation of the errors were 0.18 mm and 0.10 mm, respectively, and the maximum error was 0.36 mm.

We analyzed the *Y*-axis control signals using the same method. Due to the substantial EMI (Electromagnetic Interference) noise produced by a nearby generator, these errors were more extensive than those of the *X*-axis control signals. In the static waiting state, the mean and standard deviation of the errors were 0.05 mm and 0.04 mm, respectively, and the maximum error was 0.21 mm. In the movement control state, the mean and standard deviation were 0.16 mm and 0.11 mm, respectively, and the maximum error was 0.42 mm. The errors produced during movement control exhibited a pattern similar to that of the *X*-axis control errors. The errors start in the same direction as the light source’s movement. However, as the bending displacement of the tripod does not occur when the Motorized Stage control direction is considered, the errors do not form a sine wave shape.

When the lab-scale experiments were conducted, a lot of noise occurred during the Motorized Stage control and measurement in the *Y*-axis direction compared to that of the *X*-axis. Since the external generator was running when we were controlling the Motorized Stage and measuring along the *Y*-axis direction, strong EMI noise occurred. This aspect was beyond our control and despite the noise, the error was ±0.25 mm. Table 1 presents information about the errors between the actual and measured locations of the stage.

According to the results of the laboratory-scale experiment, the errors for both movement directions were within 0.5 mm. However, considering the signals of the static waiting state when controlling movement in the *X*-axis direction, the system should yield sufficient bridge measurement accuracy if the EMI noise generated nearby is not large. Furthermore, the errors can be reduced more stably by reinforcing the tripod supporting the laser light source. Moreover, in the usage environment of the sensor, the laser light source does not move, and only the light receiver moves according to the structure’s displacement, unlike in the lab-scale experiment. Therefore, such significant errors do not occur in practical applications.

## 4. Results and Discussion of Displacement Measurement

### 4.1. Method of Bridge Safety Inspection

Finally, we applied the developed system to bridge safety evaluation and measured its displacement. The Beodeulgyo bridge in Korea (total length: 120.22 m, width: 27 m) was considered for this study, which is a medium-sized steel box girder bridge.

The safety evaluation was conducted through load tests and by analyzing the bridge’s behavior according to the load of the test vehicles with a set axle weight. Two 15-ton dump trucks loaded with sand were utilized for the test vehicles, and because general vehicles had to be completely controlled, the tests were conducted at dawn.

**Figure 7:**
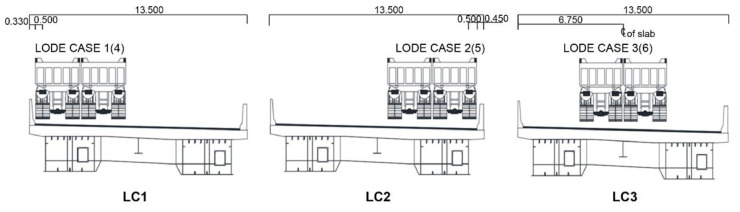
Positions of the test loading truck during the static loading test.

The tests can be categorized into static and dynamic loading tests based on the loading method. In the former, to investigate the static behavior characteristics in response to external forces, the engine of the test vehicle was turned off at the measurement point and we waited approximately a minute before measurement. Regarding the measurement positions, in principle, the load is applied to where the maximum bending moment and shear force occur. Accordingly, the test vehicle was moved to different positions for each load case (LC1, 2, 3), as shown in Figure 7.

**Figure 8:**
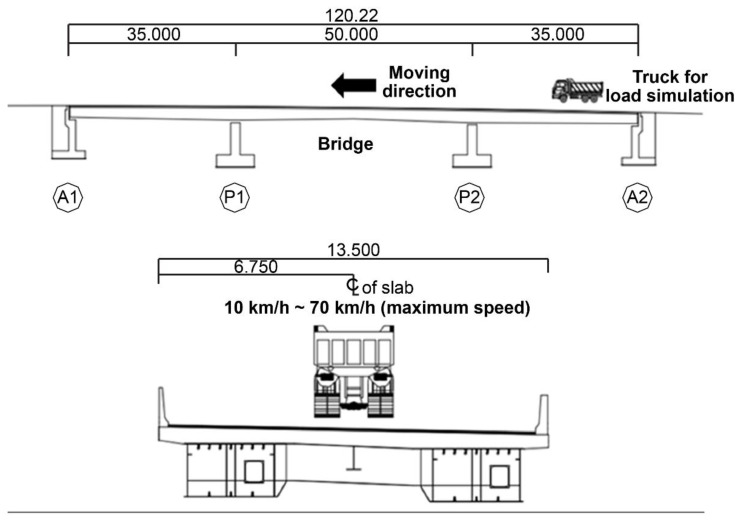
Operation process of the dynamic loading test.

A dynamic loading test was conducted to evaluate the bridge’s resistance, stiffness, and usability limitations. To investigate the dynamic behavior characteristics, the test vehicle was driven at gradually increasing speeds of 10, 20, 40, 55, and 70 km/h. The test vehicle passed the center of the bridge at a constant speed, as shown in Figure 8.

**Figure 9:**
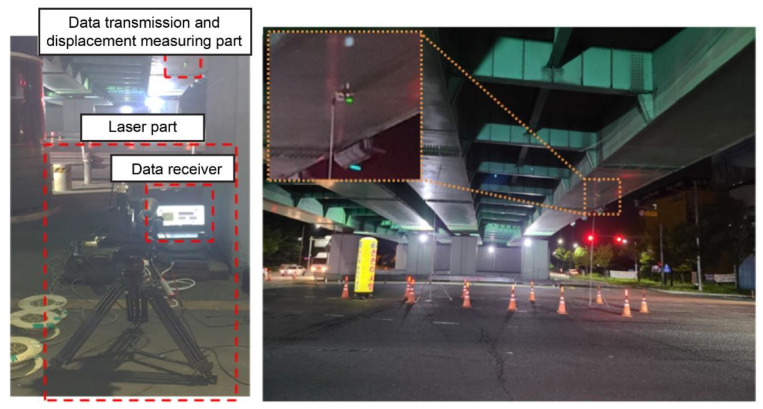
Experimental setup for the field test.

A conventional LVDT was installed at the center of the bridge, as shown in Figure 9. The light receiver and data transmitter of the developed sensor system were installed at the same position. As in the lab-scale experiments in Section 3, the laser transmitter and data receiver were installed 50 m from the light receiver, and the automatic zero-point adjustment device enabled measurement from the sensor’s center. From the ground, the height of the light receiver was 4 m. The sampling frequency for measuring the displacement was set to 300 Hz. During the field test, vertical displacements of the bridge were measured.

### 4.2. Signal Comparison for Sensing System Validation

After measuring the deflection at the center of the bridge in each loading test, the signals of the existing and newly developed sensor systems were compared to evaluate the accuracy.

There was no noise from sunlight because the experiment was conducted in the evening. However, various noises were produced by small particles such as dust, the generator, and the light source used in the loading tests. Moreover, the signals in the field test contain noise because the laser light is not perpendicularly incident to the light-receiving surface of the PSD device, unlike in the lab-scale experiments. Frequency and median filters were applied to the measured signals to improve the SNR. For the signals of the low-speed loading (10, 20, 40 km/h) and static loading tests, the frequency-filter range was set to 0.01 to 5 Hz and the median filter factor to 100 to 500. For the high-speed loading test (55, 70 km/h), as bridge behavior occurs in the high-frequency region, the frequency-filter range was set to 0.1 to 20 Hz and the median filter factor to 5 to 10.

In the dynamic loading test, when the truck simulating the load passed the measurement point, the bridge momentarily exhibited downward followed by upward deflection due to recoil, and the cycles of this behavior shortened as the truck speed increased. Specifically, at test speeds of 55 km/h and above, the bridge’s self-vibration component was more prominent after applying the load. In the static loading test, the bridge displacement signals formed a stair-like pattern when a continuous load was applied.

**Figure 10:**
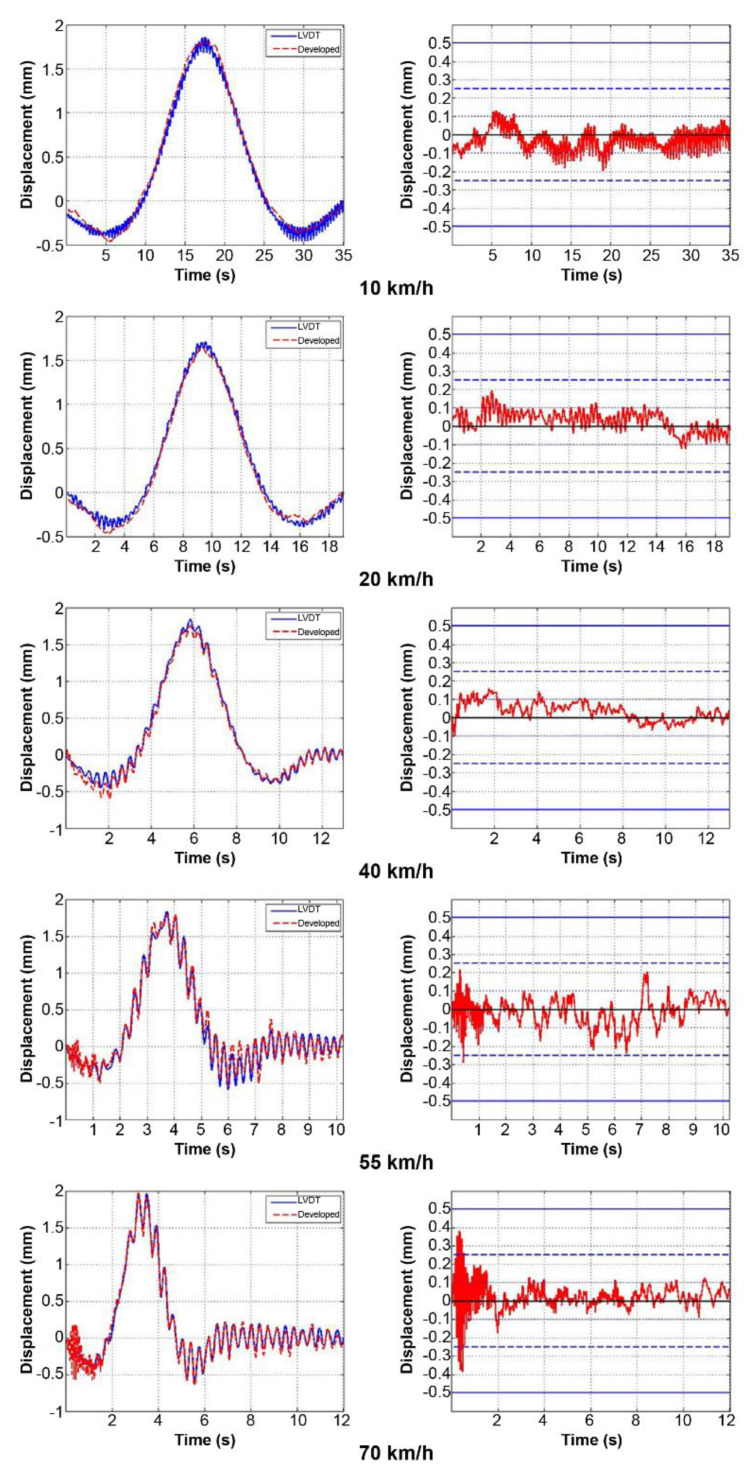
Signal comparison and errors of the dynamic loading test.

**Figure 11:**
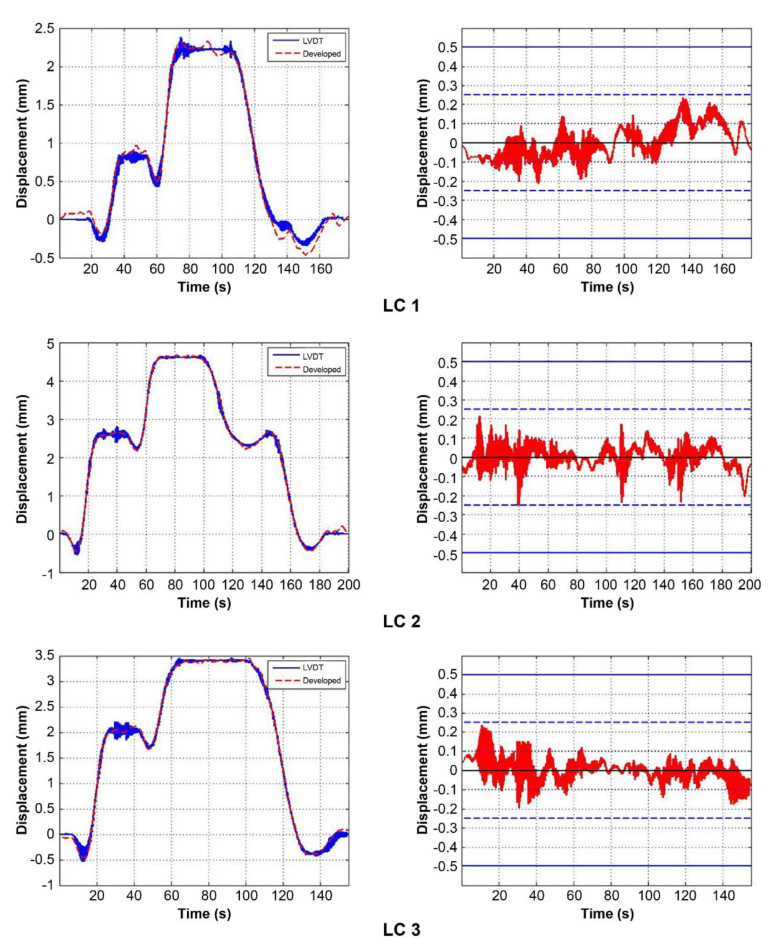
Signal comparison and errors of the static loading test.

**Figure 12:**
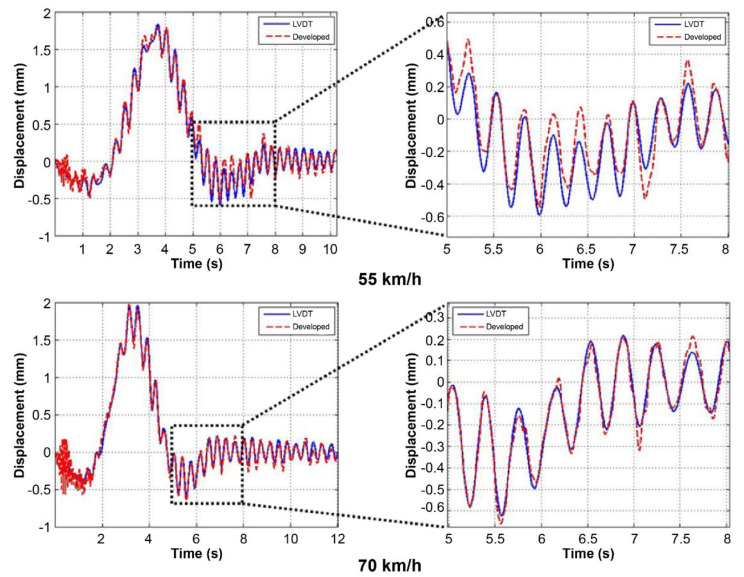
Bridge vibration motion comparison.

Figure 10 and Figure 11 show signal comparisons of the existing and developed sensors for each test, and the results show that the developed system can measure bridge displacement with high accuracy. Figure 11 shows expanded signal comparisons at 55 and 70 km/h, where the bridge’s self-vibration is prominent. As demonstrated in Figure 12, the developed system can accurately measure the bridge’s overall displacement and self-vibration, which is relatively small and occurs in the high-frequency region.

This study also analyzed the errors of the developed sensor signal compared to that of the existing one, as shown in Table 2. For the dynamic loading test, we analyzed the errors at the points with the maximum load. The average error of the signals overall was less than 0.07 mm, and the standard deviation was less than 0.05 mm. Moreover, a comparison of values where bridge displacement was most significant in the dynamic loading test showed that the error was less than 0.1 mm except for the second signal (20 km/h). The error was highest at 20 km/h, but gradually decreased as further tests were carried out. This was considered to be due to noise caused by external factors beyond our control, such as EMI noise. Furthermore, it could be regarded that the amplitude of the error was not related to the speed of the test vehicle because even if the speed was high, the resulting bridge behavior was slow enough to be measured using our Wi-Fi module. In addition, instability induced by the vehicle speed did not affect the development sensor system. Comparing the signal comparison and error analysis results, it is evident that the developed sensor system can accurately and precisely measure the bridge displacement.

## 5. Conclusions

This study developed a laser-based bridge safety diagnosis system for the remote measurement of bridge displacement. The system primarily comprises a laser beam transmitter and a light receiver, and uses a PSD as the light-receiving element. The light receiver was attached to the measurement point on the bridge and wirelessly communicated information about the laser beam’s position irradiated to the light-receiving surface and then to the PC (displacement measuring part). To verify the developed system, we first conducted lab-scale experiments and then applied the system to loading tests using a bridge for the field tests.

Based on the application results, the system can accurately measure the bridge deflection and vibration behavior according to the bridge characteristics under various load conditions. According to the error analysis results, the average error was less than 0.07 mm, and the maximum error in the dynamic loading test was less than 0.1 mm in most cases.

The system enables precise measurement even from a long distance, has a simple structure, and is robust against various noise factors. Furthermore, because it can be quickly installed and disassembled, these tasks do not require extensive labor and long periods of traffic control.

Meanwhile, the developed sensor system has various limitations due to the installation of the light receiver at the measurement point. First, because the part must be located at the measurement point, it is impossible to measure if the structure is too far away or difficult to access. Moreover, high-speed sampling is complex because the displacement data measured by the light-receiving unit must be transmitted through wireless communication, and there are issues with continuous power supply and management. However, because the measurement target is a large structure (such as a bridge), and an installation location is where existing sensors can be installed (such as under a bridge), these limitations can be overcome through additional research.

Future studies can further enhance the sensor system’s performance by introducing concentrated laser designs, implementing designs robust against EMI noise, and reducing vibrations in the laser light source support. Moreover, by simplifying and improving the light receiver’s structure, future systems using multiple light receivers can simultaneously measure multiple points.

## Figures and Tables

**Table 1:** Means errors, standard deviation errors, and maximum errors for measured displacement signals during the lab-scale experiments.

Signal	Mean Error (mm)	Standard Deviation Error (mm)	Maximum Error (mm)
*X*-axis static range	0.01	0.01	0.03
*X*-axis dynamic range	0.18	0.10	0.36
*Y*-axis static range	0.05	0.04	0.21
*Y*-axis dynamic range	0.16	0.11	0.42

**Table 2:** Mean errors, standard deviation errors, and maximum errors for measured displacement signals during the field tests.

Test Case		Mean Error (mm)	Standard Deviation Error (mm)	Error at the Maximum Load (mm)
Dynamic loading	10 km/h	0.06	0.04	0.04
	20 km/h	0.05	0.03	0.12
	40 km/h	0.05	0.03	0.08
	55 km/h	0.06	0.05	0.05
	70 km/h	0.05	0.05	0.05
Staticloading	LC1	0.07	0.04	
	LC2	0.04	0.04	
	LC3	0.04	0.04	

## Data Availability

Not applicable.
